# Correspondence

**Published:** 1896-12

**Authors:** 


### CORRESPONDENCE.

WASHINGTON, D. C., November 24, 1896.

FELLOW-MEMBERS OF THE AMERICAN VETERINARY PROFESSION: An opportunity has been kindly offered us by the Pasteur Monument Committee of France to contribute to the memorial fund which is now being raised. No one can appreciate more truly than the veterinarian how much Pasteur did for our profession and for humanity, and no other profession can feel a deeper interest in the success of the American subscription. Up to this time the veterinarians of our country have done nothing toward this object, because the subject has not been properly brought to their attention. I now appeal to every member of our profession to contribute something to this fund. Whether it be one dollar or ten dollars, it will testify to the sentiments of the giver and will assist in swelling the funds which we hope will creditably represent the great country in which we live. All contributions should be sent by draft or money order to Dr. E. A. de Schweinitz, Secretary Pasteur Monument Committee, Cosmos Club, Washington, D. C., who will duly receipt for the same. The subscription must be closed by February, and the Paris Committee desires an estimate within a month of the amount that will be contributed in order that the work on the monument may be commenced. Will you not aid the Committee and sustain the reputation of our profession by acting promptly and liberally in this matter of international interest?

D. E. SALMON,
Chairman of the Committee on Pasteur Memorial Fund of U. S. V. M. A.

---

A NEW YORK city paper of recent date states that Dr. R. S. Huidekoper, veterinarian of the New York College of Veterinary Surgeons, was instrumental in having a warrant of arrest issued and served upon a party practising as a veterinarian in that State without the proper credentials, contrary to law. I am but a veterinary student, and, if allowed space to express my humble opinion, will say that I am glad to see one man with the undaunted courage and respect for the laws of the State and veterinary society to openly stand up and protect them and the legal veterinary profession. This also encourages the student, who after expending time and money for a collegiate education, as a general rule, graduates only to find that he has a hard and bitter fight to wage with the unscrupulous empiric of to-day.

A STUDENT.

